# Surface potential modulation as a tool for mitigating challenges in SERS-based microneedle sensors

**DOI:** 10.1038/s41598-022-19942-7

**Published:** 2022-09-23

**Authors:** Vitor Brasiliense, Ji Eun Park, Eric J. Berns, Richard P. Van Duyne, Milan Mrksich

**Affiliations:** 1 Department of Chemistry, Northwestern University, Evanston, IL-60208 USA; 2 Department of Biomedical Engineering, Northwestern University, Evanston, IL-60208 USA; 3 Department of Cell and Developmental Biology, Northwestern University, Chicago, IL-60611 USA; 4 Present Address: PPSM, ENS Paris-Saclay, CNRS (UMR 5831), Université Paris-Saclay, 91190 Gif-sur-Yvette, France

**Keywords:** Sensors, Bioanalytical chemistry

## Abstract

Raman spectroscopic-based biosensing strategies are often complicated by low signal and the presence of multiple chemical species. While surface-enhanced Raman spectroscopy (SERS) nanostructured platforms are able to deliver high quality signals by focusing the electromagnetic field into a tight plasmonic hot-spot, it is not a generally applicable strategy as it often depends on the specific adsorption of the analyte of interest onto the SERS platform. This paper describes a strategy to address this challenge by using surface potential as a physical binding agent in the context of microneedle sensors. We show that the potential-dependent adsorption of different chemical species allows scrutinization of the contributions of different chemical species to the final spectrum, and that the ability to cyclically adsorb and desorb molecules from the surface enables efficient application of multivariate analysis methods. We demonstrate how the strategy can be used to mitigate potentially confounding phenomena, such as surface reactions, competitive adsorption and the presence of molecules with similar structures. In addition, this decomposition helps evaluate criteria to maximize the signal of one molecule with respect to others, offering new opportunities to enhance the measurement of analytes in the presence of interferants.

## Introduction

Surface-enhanced Raman spectroscopy (SERS) is a highly sensitive analytical technique that can improve Raman signal of various analytes by factors as high as 10^8^ due to nanoscale high electric field enhancements in the vicinity of noble metal with nanostructures^1–3^. It holds great promise in biosensing contexts, having been employed, for example, for the detection of glucose^4,5^, neurotransmitters^6,7^, and various other biomarkers^8–10^. Inexpensive SERS platforms compatible with biosensing that enable continuous and minimally invasive detection are however still scarce. Among various sensor platforms, microneedles (MNs) have received attention as a minimally invasive sensor platform for monitoring analytes in biofluids, especially in the interstitial fluid (ISF)^11–16^. In particular, MNs have demonstrated promising results in continuous detection of important ISF biomarkers, such as glucose^13,17–20^ and lactate^12^. However, as these approaches require enzyme labelling, many efforts have been made to combine MNs with enzyme-free analytical techniques such as SERS^15,21–24^. The steep distance-dependent decay of SERS, however, limits the enhancement zone to the vicinity of the surface (~ 1 nm)^25^, leading to the essential role of surface adsorption on SERS platforms in the context of sensors^26–28^, and prompting the synthesis and design of numerous chemical strategies for enabling specific molecular recognition and fixation. Frequently, the nanostructures are functionalized with chemical receptors for the analyte, such as antibodies^29^, oligonucleotides^30^, or small molecules^4,31^. In these approaches, however, the receptor often occupies this interfacial region, causing the bound analyte to lie outside of the sensing zone and preventing direct detection. Moreover, if continuous monitoring is required, binding of the analyte must be reversible, and the association and dissociation of the analyte and receptor should have rate constants that allow rapid equilibration but still sufficient affinity to detect the analyte at relevant concentrations. Alternatively, complex degradation strategies can be designed, which require extra preparation steps, and might be difficult to implement in some situations^32–34^.

In another approach, surface potential can be used to manipulate adsorption and desorption of analytes on SERS sensors^32,35–37^. In this paper, we explore this methodology, applying electrical potential to selectively localize analytes in the interfacial region of plasmonic microneedles, where they can be detected. Our strategy is based on the observation that molecules localize to an electrode in a potential-dependent manner and that the threshold potentials required for this partitioning vary with molecular structure and redox properties^35,38^. Moreover, the interaction of polarizable molecules with a polarized surface can alter electronic structure, with consequences for molecular orientation and adsorbate stability^39–41^. Hence, by varying the potential of the sensor surface during the SERS measurement, analytes can be selectively concentrated in the nanostructure interfacial region, where the field enhancement is maximal, without the need for a receptor. By continuously cycling the surface potential, we can move different analytes in and out of the detection zone, which allows discrimination of different species in the solution. We demonstrate the effectiveness of this strategy in a biosensing relevant MN platform by detecting caffeine, a model bioanalyte, in the presence of potentially confounding phenomena, such as surface oxidation, electrolyte specific adsorption, and the presence of molecules of similar chemical structure.

## Results and discussion

### Fabrication and characterization of gold (Au) MNs

**Figure 1:**
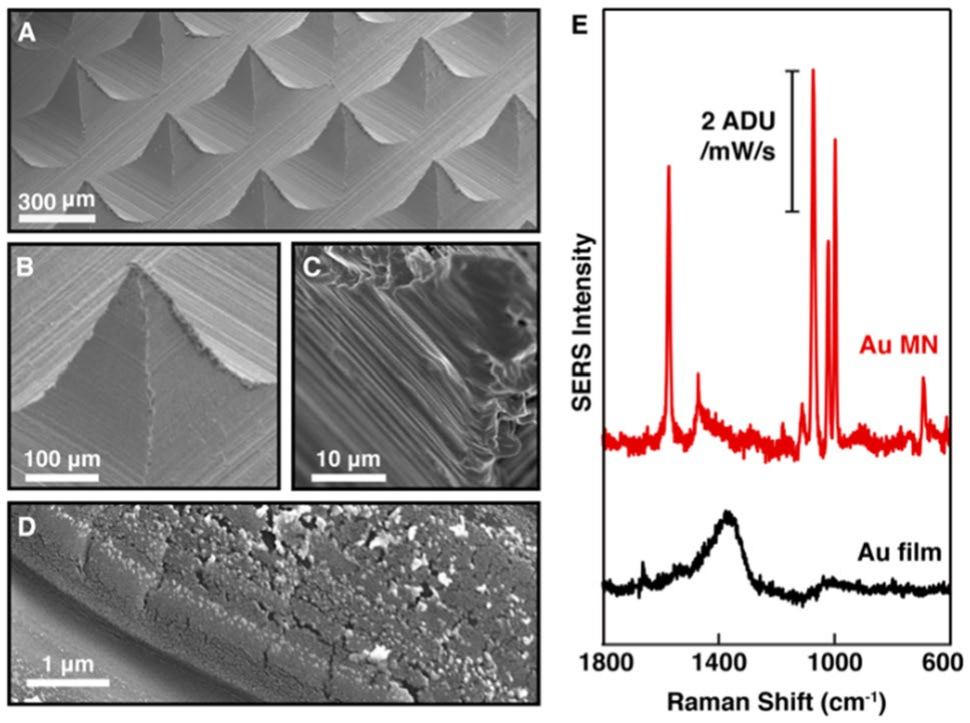
Characterization of Au-coated polymeric microneedles (Au MNs). (**A**–**D**) Characterization of the surface by scanning electron microscopy, revealing the hierarchical structure which results from the molding and gold evaporation processes. (**E**) SER spectrum of BZT functionalized on Au MNs, compared that on an Au film on a flat NOA (with an adhesion layer of titanium).

We fabricated sensors by placing Norland Optical Adhesive (NOA) prepolymer solution in a PDMS MN mold and curing it using UV light as we described previously^24^. We then coated the surface with a 5 nm adhesion layer of titanium using electron beam deposition followed by a 40 nm layer of gold using thermal deposition (Fig. 1A–D). The titanium layer significantly improves the adhesion of gold onto the polymer, enabling several (> 10) potential cycles with no noticeable degradation of the sensor. The resulting plasmonic Au MNs shows characterization of their Raman enhancement performances.

The molding and Au evaporation processes create an intrinsic hierarchical structure displaying a fine rugosity with characteristic lengths spans from a few tens to hundreds of nanometers (Fig. 1D). The intrinsic SERS performance of the substrate was demonstrated by functionalizing the surface with benzenethiol (BZT), a commonly used Raman reporter molecule, and recording the resulting SER spectra (Fig. 1E). We incubated the MNs in a 10 mM BZT ethanol solution for 45 min to form a self-assembled monolayer over the sample, saturating plasmonic hotspots. The MN sample was then mounted on the inverted microscope and the signal was recorded. While BZT functionalized Au MNs produced a sharp SERS spectrum where the characteristic BZT vibration bands can be easily observed, a control consisting of flat NOA slab with the same Au/Ti film showed no significant SERS activity. The resulting SERS enhancement of BZT signal by AuMNs was comparable to other standard SERS substrates, such as a Au film-over-nanosphere surface (Supplementary Fig. S2), therefore demonstrating the intrinsic performance of AuMNs as SERS substrates.

### Potential dependent detection of caffeine with Au MNs

To determine whether surface potential modulation on Au MNs could be used to improve detection of biomolecules, we tested device performance in solutions containing caffeine. Surface potential can influence the electronic distribution of polarizable adsorbed molecules, therefore the electrostatic interactions of organic molecules with the surface can induce conformational changes and orientations with respect to the surface^39,41^. Pyridine, for example, is known to adsorb vertically or horizontally, depending on the surface potential^39^. Caffeine is a N-containing heterocycle which shares structural similarities with pyridine*.* Therefore, we hypothesized that a similar potential-dependent orientation behavior could also occur with caffeine, leading to differences in the stability of adsorbates with surface potential modulation.

**Figure 2:**
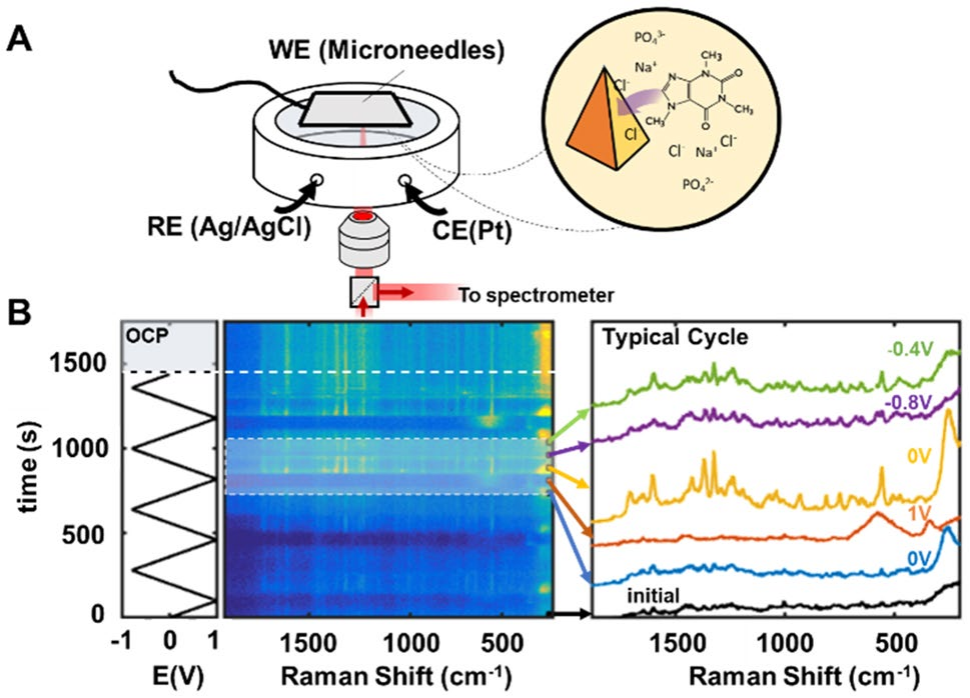
SERS response of Au MNs in PBS solution in the presence of 1 mM caffeine as a function of the surface potential. (**A**) Experimental setup sketch, and (**B**) evolution of the spectral with time and potential. The plot shows an intensity profile of the SER spectrum for each time point, where the time is related to the applied potential shown on the left. A typical cycle is highlighted, and selected spectra are shown in detail (average over six 5 s spectra), as described in the text.

To control the sensor surface potential, we used a spectro-electrochemical cell filled with phosphate buffered saline (PBS, pH = 7.4) containing caffeine (1 mM). The laser excitation beam (785 nm) was focused on the sample surface through a 20 × objective (Fig. 2A) as described in detail in the Methods section. Figure 2B shows the evolution of the SER spectra as the potential was cycled between − 0.8 and 1 V (vs. Ag/AgCl). Initially, only a very weak background was observed (Fig. 2B, black spectrum) barely exceeding noise levels except for a faint shoulder at around 260 cm^−1^, associated with Au–Cl^−^ vibration^42^.

As the potential was raised to more anodic values, the 260 cm^−1^ mode increased in intensity due to increase of the surface concentration of Cl^−^. At approximately 0.7 V, a broad band at ~ 560 cm^−1^ together with a smaller band at 350 cm^−1^ associated with gold oxide (AuO_x_)^43^ starts to build up and dominates the spectrum until the potential was lowered.

Due to AuO_x_’s poor plasmonic properties, any other Raman bands potentially present remains silent as long as surface oxide can be detected. Once the potential was lowered below 0.7 V, the 260 cm^−1^ band becomes progressively more visible again, and caffeine characteristic vibration bands appeared^44^. At more cathodic potentials (E < − 0.4 V), the intensity of most bands decreased, and only the background was detected, presumably due to caffeine desorption and formation of a double layer dominated by cations (Na^+^ and K^+^), whose adsorption on bare Au show no apparent Raman signal. Interestingly, caffeine bands seem to appear more strongly in the return trace, and the intensity of these bands was stronger after the initial cycles. The reason for these observations may be due to one or more factors. The stronger bands in the return cycle and the improvement at later cycles might be due to the presence of residual gold oxide, resulting from the brief application of cathodic potential (> 0.7 V vs. Ag/AgCl). A beneficial effect from oxide formation improving the affinity between the surface and the analytes has recently been reported^45–47^. Alternatively, some degree of surface roughening might be present, although ex-situ SEM imaging taken after 10 redox cycles did not reveal any appreciable surface modification. However, due to the intrinsic surface heterogeneity of SERS substrates, it is difficult to completely rule out any contribution of surface roughening.

The contribution of each of these chemical species can be further discriminated with the help of multivariate methods, such as Principal Components Analysis (PCA), which decompose the dataset in new representations (loadings or eigenvectors)^48–50^. Such procedure is especially appealing in the case of spectroscopic data, as the extracted eigenvectors can be regarded as spectra on their own, enabling their interpretation in terms of the chemical species present^36,51–60^. While such methods are often prone to sample-to-sample variations^52,61^, requiring extensive pre-processing of the data sets and risking the introduction of artifacts, our surface potential modulation strategy allows acquisition of a large number of spectra locally (e.g., at a given spot and sample). This enables efficient application of the multivariate analysis strategy, minimizing the influence of background and other confounding factors and allowing application of PCA algorithm to minimally processed data (only cosmic rays are removed).

**Figure 3:**
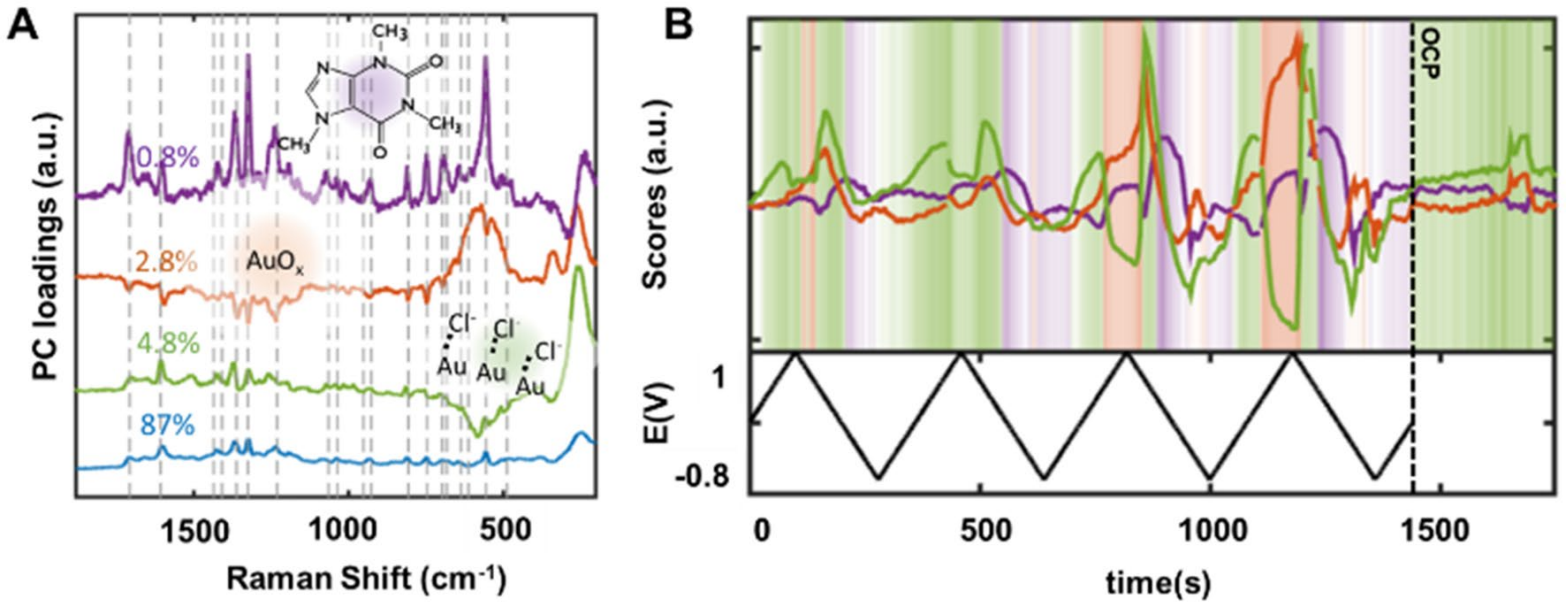
PCA on SER spectra obtained using Au MNs in PBS containing 1 mM caffeine with surface potential cycled between − 0.8 to 1 V (vs. Ag/AgCl). (**A**) The dominant PCs of the data, which account together for > 95% of the dataset dynamics (gray dashed lines represent known characteristic caffeine vibrations^39^). (**B**) Dynamics of the 2–4th PCs intensity compared to the potential over time. The shading zones indicate the dominant PC and the intensity of the background colors is proportional to the ratio between the maximal PC intensity and the sum of the other (2–4) PCs, following the colors indicated in the structures. After the end of the experiment (t − 1450 s), the system is left at open circuit potential (OCP) for $\sim$ 150 s.

Figure 3 shows the main PCs (Fig. 3A) extracted from the previous dataset next to the evolution of their time traces (Fig. 3B). As expected, the first principal component (PC) (Fig. 3A, blue trace) reflects only global characteristics of the dataset (Supplementary Fig. S3), which is not useful for identifying analytes. PC2 is dominated by the Au–Cl^−^ vibration band at 260 cm^−1^ (Fig. 3A, green trace) and PC3 by a broad mode at ∼ 560 cm^−1^ (Fig. 3A, orange trace), corresponding to AuO_x_, as previously indicated. Comparison of the PC time traces with the applied potential also support this interpretation, as the Au–Cl^−^ adsorption PCs are dominant over E > 0.3 V, in agreement with Cl^−^ adsorption potential over gold^62^, while PC3 becomes dominant at applied potentials greater than 0.7 V due to AuO_x_ formation, inhibiting SERS performance, and decreasing the intensity of all other bands (and consequently the coefficients of all other PCs). Although discrete in the first two cycles, this effect becomes more pronounced from the third cycle on, due to facilitated oxide formation in the presence of residual oxide and surface defects. The PC4 (Fig. 3A, purple trace) contains all caffeine characteristic vibration bands, similar to caffeine bands previously reported^44^ and to bands observed in reference SERS spectra (Supplementary Fig. S4). Furthermore, its time trace is consistent with previously observed caffeine adsorption behavior, becoming dominant over the other PCs in the return trace of the voltammogram after formation of AuO_x_ until more cathodic potentials (E < − 0.4 V). Taken together, these first four PCs account for over 95% of the total data variance, and therefore characterize the majority of the dataset dynamics. Attempts to improve the separation ability of the PCA procedure by pre-processing the data with smoothing filters and background subtraction introduced artificial correlations in the data, worsening the separation.

We note that since the spectral representation yielded by the PCA algorithm is extracted from correlation data, it is critical to record numerous adsorb-desorb events, highlighting the importance of being able to modulate the sensor spectral response cyclically. We tested the effect of having less data available for analysis (Supplementary Fig. S5), which resulted in less well-defined separation and poorer correlation between the temporal evolution of the PCs and the chemical events (e.g., adsorption, redox reactions). Analysis of the complete data set showed a significant improvement in the signal-to-noise ratio due to a better segregation of noise to higher order PCs. We therefore show that nonspecific electrolyte adsorption and surface oxidation, common challenges in biosensing, can be significantly minimized through electrochemical surface potential modulation in association with multivariate analysis.

### Discrimination between similar chemical species

The results of the previous study demonstrate the ability of this surface modulation approach to separate the contribution of chemical phenomena with different dynamics (e.g., adsorption of molecules of different sizes, surface redox reactions). It raises the question of whether molecules with similar chemical structures can also be discriminated.

**Figure 4:**
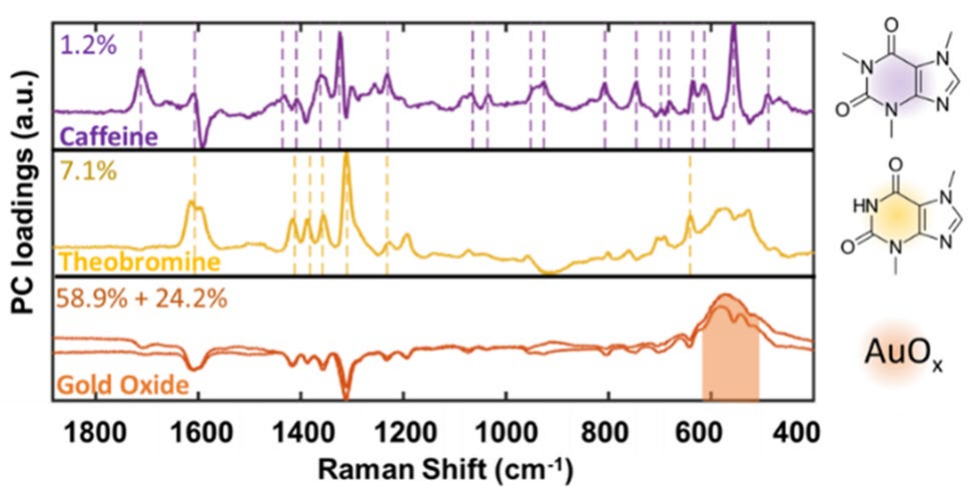
Eigenvectors extracted from a total of 700 spectra (t_total_ = 3500 s) accounting for 91.4% of the dataset dynamics. The presence of caffeine peaks (purple dashed lines) on the 4th PC (purple) can be clearly observed, while the 3^rd^ PC shows theobromine vibration bands (yellow dashed lines), and the 1st/2nd PC (orange) primarily shows AuO_x_ vibration.

To test this, we repeated the experiment in the presence of an equal concentration (1 mM) of an important caffeine metabolite, theobromine, which differs from caffeine by only one methyl group (Fig. 4). A large dataset (N = 700 spectra) was collected by cycling the mixture solution between − 0.8 and 1 V for 10 times at 10 mV/s. The results are consistent with the previous experiment, with appearance of AuO_x_ at very anodic potentials (E > 0.7 V), potential dependence for the appearance of caffeine peaks at intermediate potentials (0.7 V > E > − 0.4 V), and desorption at very cathodic potentials (E < − 0.4 V). However, the potential range at which caffeine and theobromine adsorbed onto the substrate was similar, posing a significant challenge to manual separation of the contributions of caffeine and theobromine.

**Figure 5:**
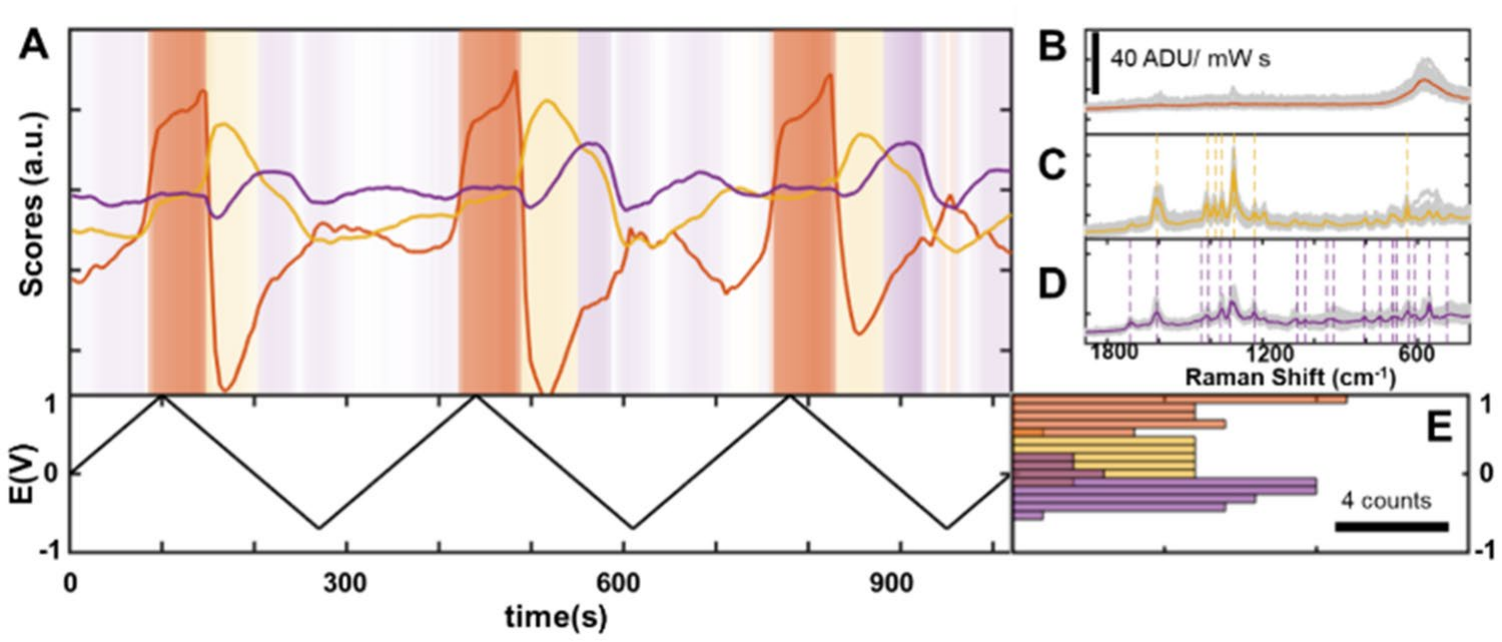
(**A**) Evolution of the potential and PC scores with time over three potential cycles between -0.8 and 1 V. The intensity of the background colors is proportional to the ratio between the maximal PC intensity (orange for AuO_x_, yellow for theobromine, and purple for caffeine) and the sum of the other PCs. (**B**) Spectra in which the AuO_x_ related PCs are dominant (gray) and their average spectrum (orange). (**C**) Spectra in which the theobromine related PCs are dominant (gray) and their average spectrum (yellow). (**D**) Spectra in which the caffeine related PCs are dominant (gray) and their average spectrum (purple). (**E**) Histogram summarizing the potential at which each PC becomes dominant (beyond a 0.1 threshold), clearly showing the dependency of the adsorption probability with the surface potential.

In contrast, multivariate analysis produces PC eigenvectors that separate the contributions of caffeine and theobromine (Fig. 4). For simplicity, the spectral region is limited to $\nu$ > 400 cm^−1^ avoiding the Au–Cl^−^ region. While PCs 1 and 2 are dominated by AuO_x_ response and its characteristic broad band at ~ 560 cm^−1^, PC3 mainly contains sharper peaks, corresponding to characteristic bands of theobromine^63,64^ (Fig. 4, PC3, yellow), and caffeine (Fig. 4, PC4, purple). These results demonstrate that the absorption potential dependencies of molecules with similar structures is sufficient to enable their spectral discrimination, as all main peaks from theobromine and caffeine were successfully extracted and separated without any need of providing standards or reference spectra. A more detailed analysis shows the evolution of the coefficients of the main PCs with the potential over three potential cycles (Fig. 5A). Since the first two PCs account for the presence of AuO_x_, we have clustered them together by summing their scores. This PC coefficient becomes dominant at anodic potentials, concomitantly with the appearance of anodic current and consistent with the previous experiment (Figs. 2 and 3).

The average spectrum of all frames in which this PC becomes dominant clearly portrays the characteristic broad band of AuO_x_ (Fig. 5B). Similarly, averaging the SER spectra for each dominant component (PC3 in Fig. 5C and PC4 in Fig. 5D) allows clear identification of all characteristic vibrations from theobromine and caffeine. Interestingly, we once again observe that caffeine and theobromine characteristic bands only appear on the return trace of the voltammogram, after AuO_x_ is formed and reduced, which again suggests the role of small amounts of oxide in the adsorption of both theobromine and caffeine^45–47^.

Surface potential criteria for selectively localizing each species in the SERS hot-spots can be derived by considering the potential at which each PC becomes dominant over the others, as shown in the histograms of Fig. 5E. One can clearly identify the anodic region where the AuO_x_ (first two PCs) dominates the SERS response, and also the spectral regions where caffeine is more likely to adsorb with respect to theobromine (0 > E > − 0.3 V).

This allows potential modulation strategies to be regarded as a pseudo-separative method, enabling the analysis of analytes in complex media in an automated and straight-forward manner. In the context of in vivo biosensors design, the strategy we report can be applied to various conductive plasmonic sensor platforms to enable highly sensitive and selective analysis of chemically complex media.

## Conclusions

In this work, we demonstrated how analyte-substrate affinity can be modulated with the help of surface potential on a MN biosensor platform. Coupled with multivariate analysis methods such as PCA, this strategy enables discrimination of spectral responses between molecules with similar structures and extraction of information about the surface-analyte interaction dynamics. Specifically for caffeine, we have demonstrated that surface potential modulation can be used to cope with different commonly observed problems in biosensing contexts, such as specific electrolyte binding, surface oxidation, and presence of molecules of similar structure to the target analytes. We also show that surface modulation and in situ operando analysis enable the development of criteria for favoring detection of one analyte over the other. Finally, although the reported results are presented in the context of microneedles, this receptor-free strategy requires only a conductive sensor surface, therefore being useful in a much broader context of SERS sensing.

## Methods

### Chemicals and materials

Benzenethiol (BZT), caffeine, theobromine, and 1 × phosphate buffered saline (PBS) tablets (pH 7.4) were purchased from Sigma-Aldrich and used without further purification. Milli-Q water (18.2 MΩcm) was used in all preparation. Gold pellets (99.999% pure) were purchased from Kurt J. Lesker. Norland Optical Adhesive (NOA) 65 was purchased at Norland Product Inc. Microneedle PDMS molds (pyramidal features with 300 µm height, 200 µm base, and 500 µm tip-to-tip distance in a 25 × 90 array) were purchased from Micropoint Tech, Singapore. Hot melt adhesive was purchased from Surebonder.

### Instrumentation

Scanning electron micrographs (SEM) were taken with Hitachi SU 8030, operating at an acceleration voltage of 5–10 kV. NOA prepolymer solution was cross-linked at 365 nm wavelength using UVP CL-1000L. The SERS experiments were conducted with an inverted microscope (Nikon TE300) with a 20 × extra-long working distance objective (Nikon, NA = 0.45). A continuous wave diode laser beam ($\lambda$ = 785 nm, Innovative Photon Solutions) was focused on the sample and recollected by the same objective and passed through a 785 nm long-pass filter (Semrock) to eliminate Rayleigh scattering and directed to the Raman spectrograph equipped with a liquid nitrogen-cooled CCD detector (Action 300i, Spec-10) and a 600 grooves/mm grating. We limited the power focused on the sample to 1mW in order to avoid side reactions and parasitic signal. Collected SERS spectra were processed using MATLAB. All electrochemical experiments were performed using a CH Instruments potentiostat (CHI660D).

## Data Treatment

All data was processed using MATLAB. After removing cosmic rays using a thresholding routine. Decomposition of the datasets is achieved with the built-in “pca” function.

### Au-coated polymeric microneedles (Au MNs) working electrode fabrication

NOA 65 prepolymer solution (− 1.5 g) was dispensed into a MN PDMS mold. The mold was then placed in a light protected vacuum desiccator for 10 min. The air bubbles created in the mould cavities were removed and the NOA prepolymer solution was cross-linked using 365 nm UV light (exposure for 10 min). After removing the solid MN from the mold, 5 nm of titanium adhesion layer was deposited on the surface using an e-beam deposition system followed by 40 nm Au deposition with a thermal deposition system. Finally, the MN patch was cut into small pieces and connecting wire was attached to the MN using silver epoxy. In order to avoid direct contact of the epoxy glue with the solution, the connecting wire, the contacts and the sides of the MN patch were protected with a hot melt adhesive (Surebonder). The NOA polymeric MN fabrication process is shown in Supplementary Fig. S1.

### Spectro-electrochemical cell setup

A spectro-electrochemical cell filled with either 1 mM of caffeine in PBS (pH 7.4) or a mixture of 1 mM caffeine and 1 mM theobromine in PBS (pH 7.4) was completed by placing the Au MN as a working electrode, a ring-shaped Pt wire as a counter electrode, and a leakless Ag/AgCl reference electrode (Harvard Instruments). For the concentration of caffeine, 1 mM caffeine was used to demonstrate the sensing ability of Au MNs. Although there is limited information on the concentration of caffeine in the ISF, Caffarel-Salvador et al. detected the highest mean caffeine concentration in human ISF to be approximately 0.5 mM after 1–2 h post consumption of Proplus tablets (total of 100 mg caffeine) using swellable MNs^65^. However, since the mean daily uptake of caffeine for all U.S. adults is reported to be about 3 mg/kg body weight^66^, corresponding to 210 mg for a 70 kg person, we chose 1 mM of caffeine to detect.

### Preparation of AuFON substrates

Si wafer disks with 25 mm diameter were cleaned by placing the wafers in Piranha solution (3:1 H_2_SO_4_:H_2_O_2_) for 1 h and rinsing with Milli-Q water. The wafers were treated with base by sonicating in 5:1:1 H_2_O:H_2_O_2_:NH_4_OH for 1 h and rinsed with Milli-Q water to make hydrophilic wafer surface. The wafers were then incubated in Milli-Q water for storage. SiO_2_ microspheres (diameter of 390 nm) were cleaned by centrifugation at 5000 rpm for 5 min and washing with Milli-Q water (3 cycles). The SiO_2_ solution was diluted to 5% by volume, drop-casted (14–15 µL) onto a cleaned Si wafer, and air-dried. Au was then thermally deposited (150 nm thickness) on the substrate at a rate of 1 Å/s under ~ 10^–7^ Torr (PVD-75, Kurt J. Lesker).

### Preparation of Au nanospheres (AuNS) for reference spectra

Reference spectra for caffeine and theobromine were recorded by dropcasting 10 µL of 90 nm citrate-stabilized AuNS (0.25 mg Au/mL, STA Technologies Inc.) on a quartz microscope slide and added 10 µL of 1 mM of either caffeine or theobromine in PBS (pH 7.4). The SER spectra were recorded with a 785 nm laser focused on the mixture droplet (parameters for data acquisition: *λ*_ex_ = 785 nm, 20 × ELWD objective, but with 1 min. acquisition time (*P* = 660 μW).

### Functionalization of Au MNs and AuFON with benzenethiol

The surface of Au MNs was functionalized with benzenethiol by incubating the substrate in 10 mM benzenethiol in ethanol for 45 min and then thoroughly rinsing with ethanol.

## Supplementary Information

Supplementary Information.

## Data Availability

The datasets generated and analyzed during the current study are available from the corresponding author on reasonable request.
