# Supplementary material for: Surface potential modulation as a tool for mitigating challenges in SERS-based microneedle sensors
Source: Sci Rep. 2022 Sep 23;12:15929. doi: 10.1038/s41598-022-19942-7 (PMC9508330; doi:10.1038/s41598-022-19942-7)
Supplement: Supplementary file 1 — Supplementary Information. [file 41598_2022_19942_MOESM1_ESM.docx]

Supplementary Information

**Surface potential modulation as a tool for mitigating challenges in SERS-based microneedle sensors**

Vitor Brasiliense^a,b,#^, Ji Eun Park^a,#^, Eric J. Berns ^c^, Richard P. Van Duyne^a,c,^†, Milan Mrksich^a,c,d,^*

*^#^ Vitor Brasiliense and Ji Eun Park contributed equally to this work.*

*^a^Department of Chemistry, Northwestern University, Evanston, IL-60208, USA*

*^b^ Current Address: PPSM, ENS Paris-Saclay, UMR CNRS 8531, Université Paris-Saclay Gif S. Yvette 91190, France*

*^c^Department of Biomedical Engineering, Northwestern University, Evanston, IL-60208, USA*

*^d^Department of Cell & Developmental Biology, Northwestern University, Chicago, IL-60611, USA*

*Vitor Brasiliense and Ji Eun Park contributed equally to this work.*

†*Richard P. Van Duyne deceased in July 28, 2019.*

**E-mail:* [*milan.mrksich@northwestern.edu*](mailto:milan.mrksich@northwestern.edu)


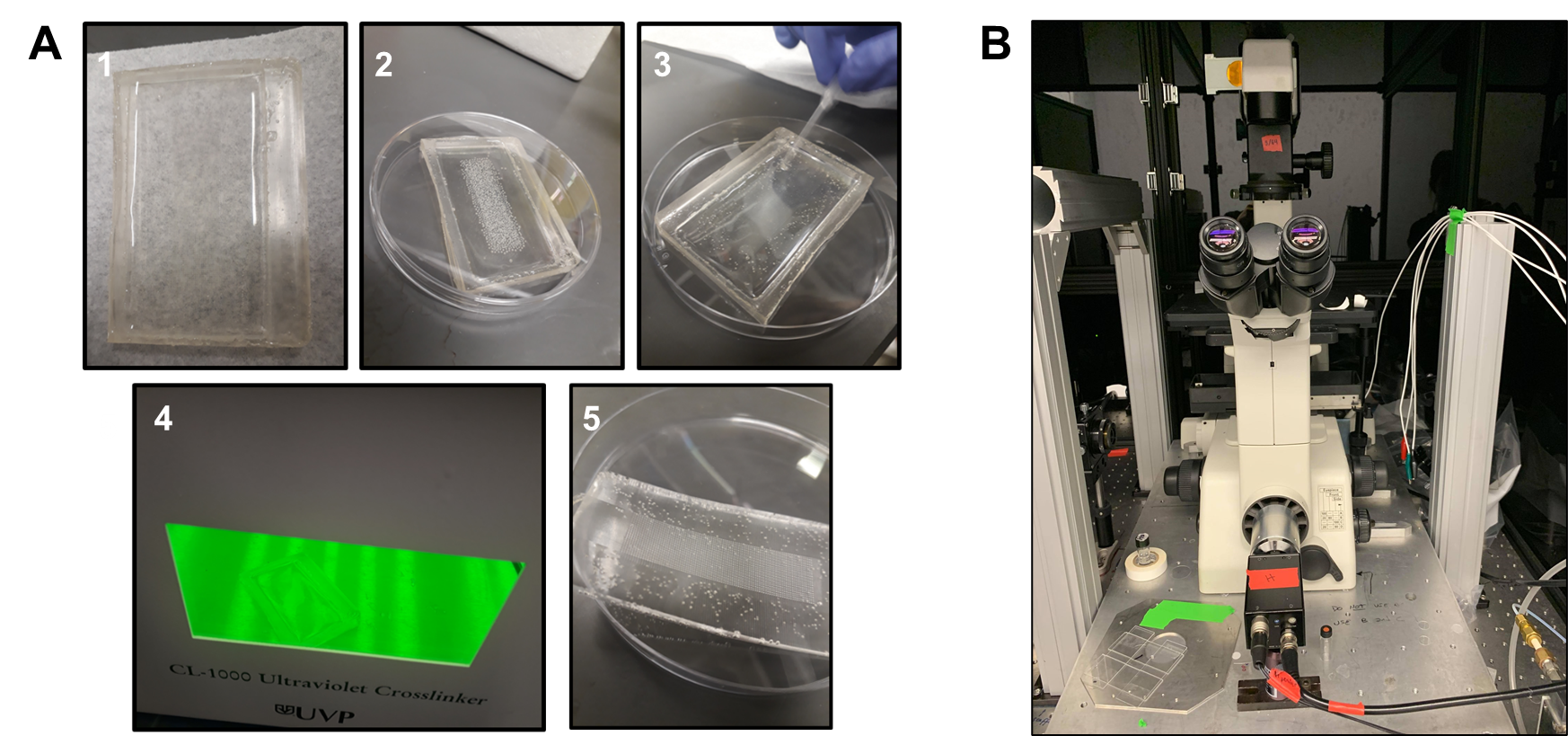


**Figure S1.** (A) Fabrication process of NOA polymeric microneedles (MNs). Step 1**)** NOA prepolymer solution was spread in the MN mold. Step 2) The mold with prepolymer solution was placed in a plastic vacuum desiccator. Step 3) After taking out the sample from the desiccator, bubbles were removed using a pipette tip. Step 4) The sample was then placed in a UV chamber to cure the polymer. Step 5) Cured MNs were taken out from the mold. After these steps, 5 nm of titanium and then 40 nm of gold are deposited onto the sensor using e-beam and thermal deposition, forming the Au MN. The Au MN is then cut into smaller samples (~20 mm^2^), and connecting wires are connected to the gold surface using silver epoxy. Direct exposure of the connecting wire to the solution is prevented by applying a layer of hot melt adhesive (Surebonder). The Au MN sensor is then loaded into a spectro-electrochemical cell and positioned in (B) an inverted microscope (Nikon TE300) equipped with a 20x extra-long working distance objective (Nikon, NA = 0.45).

**
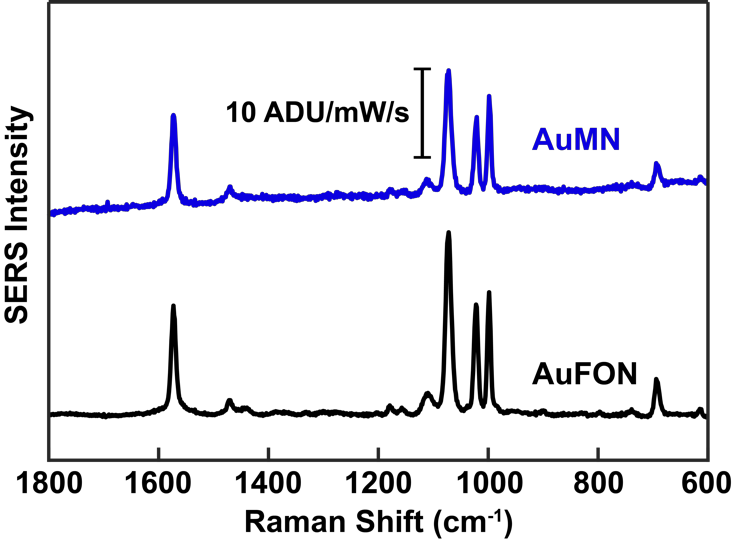
**

**Figure S2.** Comparison of SERS performance of AuMN and Au film-over-nanospheres (AuFON), a well-established SERS substrate. Both substrates were functionalized with benzenethiol as a Raman reporter. The parameters for data acquisition were λ_ex_ = 785 nm, 20x ELWD objective, t_acq_ = 1 min, and P_ex_ = 1mW.

**
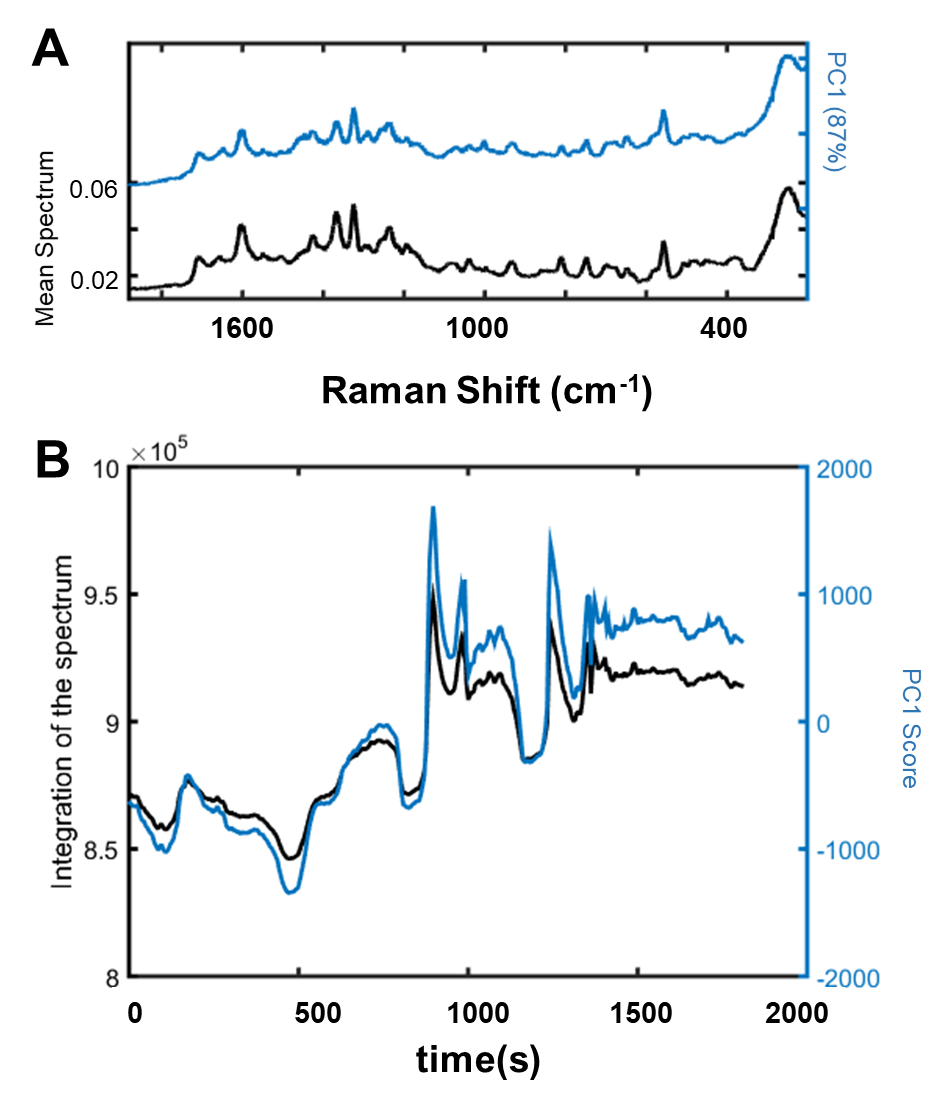
**

**Figure S3.** Analysis of the first principal component. (A) comparison with the first principal component with the mean spectrum over the full dataset. (B) Comparison of the time evolution of the first principal component score with the integration of the full spectrum. These results indicate that the first principal component describes global aspects of the dataset, and therefore is not very useful to identify analytes.


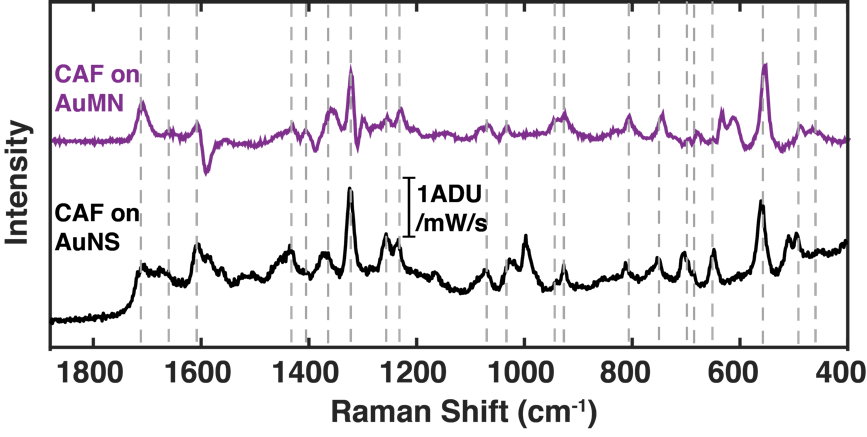

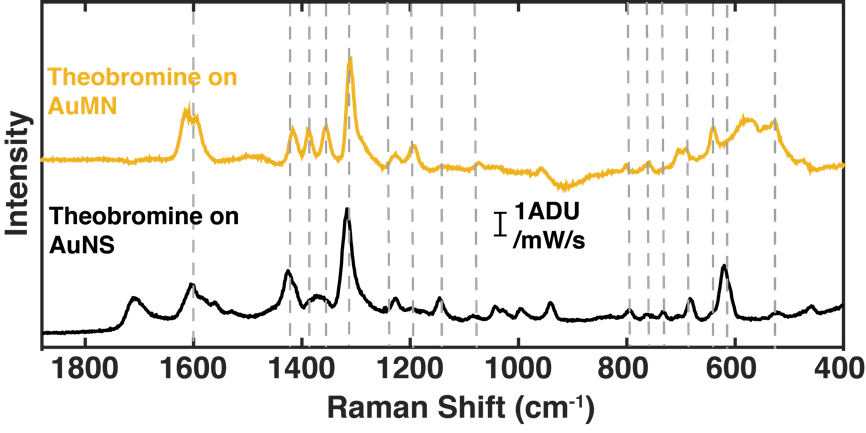


**Figure S4.** (Left) Reference SER spectra of caffeine (CAF) on AuNS (black) compared to the PC with caffeine feature where the measurement was done on AuMN (purple). (Right) Reference SER spectra of theobromine on AuNS (black) compared to the PC with theobromine feature where the measurement was done on AuMN (yellow).


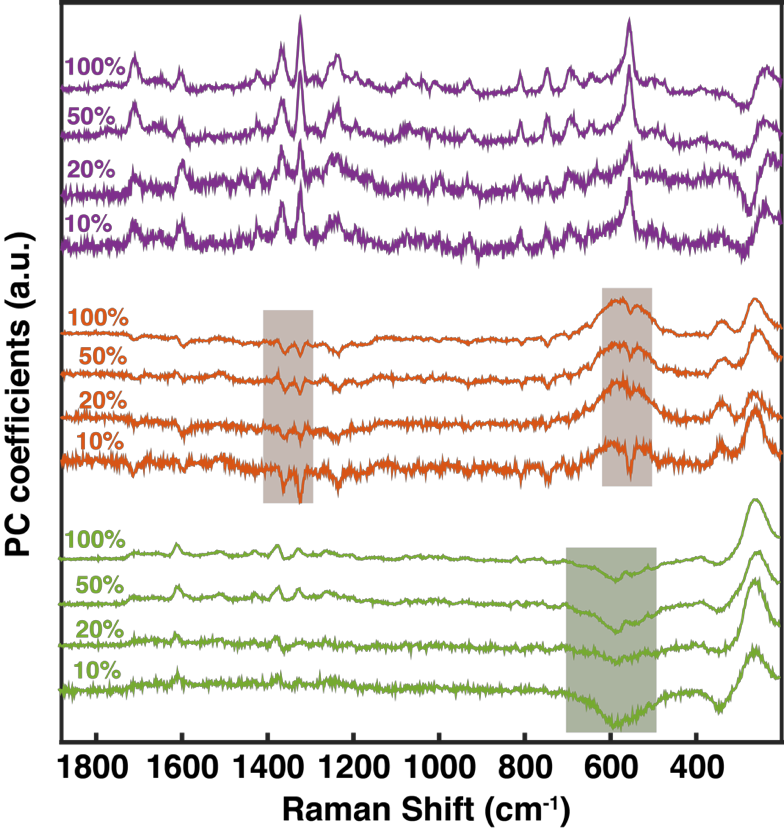


**Figure S5.** The effect of the number of spectra used in PCA. Starting from a set of 350 spectra (100%) taken at different potentials, we restricted the PCA analysis to randomly selected sub-sets consisting of 175 (50%), 70 (20%), and 35 (10%) spectra. Besides the increase in the signal-to-noise ratio, we observe better separation as highlighted by the shaded regions.
